# Assessing Discriminant Validity through Structural Equation Modeling: The Case of Eating Compulsivity

**DOI:** 10.3390/nu16040550

**Published:** 2024-02-16

**Authors:** Anna Panzeri, Gianluca Castelnuovo, Andrea Spoto

**Affiliations:** 1 Department of General Psychology, University of Padua, 35131 Padova, Italy; 2 Clinical Psychology Research Laboratory, IRCCS Istituto Auxologico Italiano, San Giuseppe Hospital, 28824 Verbania, Italy; 3 Department of Psychology, Catholic University of Milan, 20123 Milan, Italy

**Keywords:** food addiction, eating compulsivity, obesity, assessment, discriminant validity, structural equation modeling, psychometrics

## Abstract

Food addiction (FA) and disordered eating behaviors related to obesity are gaining attention in clinical and research fields. The modified Yale Food Addiction Scale 2.0 (mYFAS2.0) is the gold standard questionnaire to measure FA, while another tool is the Measure of Eating Compulsivity 10 (MEC10). Discriminant validity is present when two measures of similar but distinct constructs show a correlation that is low enough for the factors to be regarded as distinct. However, the discriminant validity of these measures has never been tested. Through a cross-sectional study design, 717 inpatients (females: 56.20%, age: 53.681 ± 12.74) with severe obesity completed the MEC10, Binge Eating Scale (BES), and mYFAS2.0. A structural equation model (SEM) was fitted, freely estimating latent correlations with 95% confidence intervals (95% CI). The results confirmed the scales’ excellent psychometric properties. Importantly, latent factor correlations between MEC10 and mYFAS2.0 (est = 0.783, 95% CI [0.76, 0.80]) supported their discriminant validity. In contrast, the latent correlation of MEC10 and BES (est = 0.86, 95% CI [0.84, 0.87]) exceeded the recommended thresholds, indicating the absence of discriminant validity and suggesting a potential overlap, consistent with previous evidence. In conclusion, MEC10 demonstrates excellent psychometric properties but is more a measure of BED and not FA.

## 1. Introduction

Among the physical conditions and diseases that have been on the rise for several decades, obesity stands out as one of the most widespread and severe clinical conditions worldwide [1]. It contributes to a higher severity of physical conditions, increased comorbidities, worse prognosis, deteriorated psychological conditions, reduced life expectancy, and imposes significant costs on national health services [2].

At the same time, the proliferation of fast foods and the ready availability of highly palatable foods have surged in recent years. Hyper-processed foods are increasingly recognized as potential triggers for the development of real addictions to these foods. Indeed, scientific literature has extensively documented that foods such as chocolate, pizza, fries, pasta, and milkshakes can activate addiction mechanisms [3].

### 1.1. Food Addiction

In line with this background, over the past 20 years, research has focused on the construct of food addiction (FA) based on the premise that certain types of food, defined as highly palatable, can activate neural mechanisms related to substance use [4]. Additionally, FA exhibits psychological behaviors typical of substance use dependence (SUD). Indeed, FA has a dual nature encompassing both substance use (highly palatable foods) and eating disorders. Indeed, episodes of binge eating are shared by FA, binge eating disorder (BED), and bulimia nervosa. Individuals with overweight/obesity and/or eating disorders (e.g., BED) are more likely to also have a FA [5,6,7,8], as they often display symptoms and behaviors that are also typical of addiction-related disorders, including both SUD and behavioral addictions [9,10,11,12,13]. Examples of these symptoms include a feeling of loss of control once eating begins [14], feeling unable to stop overeating despite an awareness of its adverse consequences [10,13], spending a huge amount of time thinking about food and/or obtaining highly palatable foods [12,13,15], and experiencing craving symptoms [16]. Consequently, all these symptoms can lead to significant impairment in different areas, including physical, social, and psychological aspects [17,18].

The scientific literature has provided some controversial evidence regarding the extent of overlap between FA and binge eating behaviors, which are typical of binge eating disorder (BED) [19]. However, meta-analytic evidence has indicated that FA and binge eating are distinct constructs [20]. Notably, binge eating is only one of the possible components of FA but not a necessary feature. The most used self-report scale to measure binge eating episodes and behaviors is the Binge Eating Scale (BES) [21].

### 1.2. Measures of FA: Overlap and Differences

The need for accurate measurement of the FA construct emerged and has led to the development of various assessment tools. To this extent, Gearhardt et al. developed the YFAS [22] and its subsequent versions, YFAS2.0 [23] and mYFAS2.0 [24]. Several studies have extensively tested the psychometric properties of the YFAS, which has been translated into multiple languages, establishing it as the gold standard tool to measure FA [25].

YFAS2.0 and mYFAS2.0 measure the 11 criteria of SUD [26], which are as follows: (i) consuming more substance than intended; (ii) inability to cut down or stop; (iii) great time spent on the substance; (iv) important activities given up; (v) use, despite adverse psychophysical problems; (vi) tolerance; (vii) withdrawal; (viii) use, despite interpersonal/social issues; (ix) failure in role obligations; (x) use in physically hazardous situations; and (xi) craving or urge to use. Each item of the YFAS2.0 is scored on a Likert-type response scale ranging from ‘never’ (=0) to ‘every day’ (=7) and can be recoded as an ordinal score according to the thresholds to determine if that symptom/criterion is present or not (endorsed vs. not endorsed) [23]. By adding up the number of endorsed criteria, it is possible to obtain the symptom count (ranging from 0 to 11) and two categorical scores as follows: the diagnostic score (presence of FA or absence of FA), requiring the presentation of at least 2 of the 11 criteria plus the criterion of significant clinical impairment, and the level of severity of FA according to the number of endorsed criteria (0–1 = none, 2–3 = mild, 4–5 = moderate, >5 = severe).

However, recently, various psychometric assessment tools have been developed to evaluate FA by measuring eating compulsivity and binge eating behaviors. The need to overcome the limitation of the YFAS2.0, which provides only a categorical score and not a dimensional one, led to the development of another tool to measure FA on a continuum, i.e., the Measure of Eating Compulsivity 10 (MEC10) [27]. The MEC10 is a brief and psychometrically sound tool designed to measure FA through its eating compulsivity component, comprising 10 items rated on a five-point Likert-type scale.

However, the Italian validation study by Rossi et al. [28] suggests that the MEC10 is more likely to measure binge eating behaviors—typical of binge eating disorder (BED)—rather than FA. The authors explained that the MEC10 is primarily focused on eating in a voracious and uncontrolled manner, which is the hallmark of BED. This behavior is only one single facet shared by FA and BED, and the MEC10 does not assess the fundamental aspects of FA, such as abstinence, tolerance, and craving—which are typical of addictions. Consequently, the authors [28] highlighted that the construct validity of the MEC10 for FA may be misattributed, as it seems to measure BED rather than FA. Indeed, it is essential to test the discriminant validity of the MEC10 and the Binge Eating Scale (BES) in measuring symptoms of binge eating episodes. Testing of discriminant validity is one of the fundamental properties that assessment tools (e.g., self-reports and semi-structured interviews) are required to satisfy.

### 1.3. Discriminant Validity

To this extent, in the last decades, psychometrics has focused on construct validity, which comprises different types, including discriminant validity [29]. As for validity in general, discriminant validity is a property of a measurement scale rather than a property of the trait or construct or trait being measured, which exists independently of its measurement [30].

Different definitions of discriminant validity have been provided. In general, discriminant validity concerns “the degree to which two measures designed to measure similar, but conceptually different, constructs are related” [31] and “A low to moderate correlation is often considered evidence of discriminant validity”. Previously, McDonald [32] stated that discriminant validity is present when two measures of similar but distinct constructs show a correlation that is “low enough for the factors to be regarded as distinct ‘constructs’”. Recently, Rönkkö & Cho [33] proposed the following generalized definition: “Two measures intended to measure distinct constructs have discriminant validity if the absolute value of the correlation between the measures after correcting for measurement error is low enough for the measures to be regarded as measuring distinct constructs”, or, about latent correlations, “two scales intended to measure distinct constructs have discriminant validity if the absolute value of the correlation between two latent variables estimated from the scales is low enough for the latent variables to be regarded as representing distinct constructs”. There is also another quite common meaning of discriminant validity, known as the ‘known group validity’, which refers to the ability of a measure to discriminate between different groups. However, our focus here is on differentiating between constructs that are distinct yet similar.

### 1.4. On the Importance of Discriminant Validity

Assessing discriminant validity is crucial for several reasons, both in clinical and research practice and, most importantly, in the development and validation of scales.

From a metascience perspective, accurately measuring constructs in psychology, which includes considering discriminant validity as it is a facet of construct validity, is vital. Poor measurement practices contribute to the replication crisis in psychology and other disciplines [34]. As noted by Loken & Gelman [35], “Poor measurement can contribute to exaggerated estimates of effect size. This problem and related misunderstandings are key components in a feedback loop that perpetuates the replication crisis in science”. Furthermore, Gelman also claimed that the replication crisis in psychology will not be (entirely) solved by better statistics but necessarily by better measurement [34], such as by avoiding questionable measurement practices [36].

Additionally, testing discriminant validity is important to overcome the jingle-jangle fallacies [37,38]: the jingle fallacy occurs when two different constructs are considered to be the same because they have the same label; conversely, the jangle fallacy occurs when two identical or almost identical constructs are considered to be different only because their names are different.

Therefore, when developing and validating a measurement tool, it is important to evaluate its discriminant validity with similar already existing scales. Likewise, when selecting a scale, it is essential to ensure its discriminant validity; otherwise, one may inadvertently measure something different from the intended construct. For example, in clinical contexts, accurate differential diagnosis among various constructs is possible only if the measures exhibit adequate discriminant validity.

### 1.5. Assessing Discriminant Validity

Different methods exist to evaluate discriminant validity and have been used in various disciplines, such as psychology and economics. Recently, a methodological paper [33] reviewed several methods for assessing discriminant validity, including the Heterotrait–Monotrait (HTMT) ratio of the interconstruct correlations [31,39], the Fornell and Larker criterion [40], the analysis of cross-loadings from exploratory factor analysis [39], and through the estimation of confirmatory factor analysis (CFA) and structural equation modeling (SEM). Based on simulation studies, the authors [33] concluded that the best-performing methods for assessing discriminant validity are those based on CFA and SEM (SEM), utilizing latent correlations and/or model-fit comparisons. Please refer to the Methods section for further details.

### 1.6. Research Gap

Taking into account the scientific background, both from the methodological and clinical perspectives, a research gap can be identified. To date, the discriminant validity of some of the most widely used FA measures—including MEC10, mYFAS2.0, and BES—has never been tested or established. Despite these scales being undoubtedly useful and well-validated, the existence of some conceptual overlap among them highlights the importance of testing their discriminant validity. While MEC10 evaluates FA, recent validation studies [28,41] have shown that, based on observed correlations, MEC10 has a stronger association with binge eating. What would the results be when estimating these correlations through a latent factors model?

### 1.7. Aim and Research Hypotheses

Considering this background, the present research aims to test the discriminant validity of the MEC10 in relation to the BES and the mYFAS2.0.

Building upon recent studies [e.g., [28]], it was hypothesized that eating compulsivity, as measured by the MEC10, should be more closely related to binge eating behaviors, as measured by BES, than to FA, as measured by mYFAS2.0. Therefore, it is expected that at a latent level, the MEC10 will be more strongly correlated with the BES than with the mYFAS2.0. In other words, it is not expected that the discriminant validity between the MEC10 and the BES is supported, as they may measure the same construct: binge eating behaviors and related feelings.

On the contrary, the discriminant validity between the mYFAS2.0 and the MEC10, and between the mYFAS2.0 and the BES, is expected to be supported, as FA and compulsivity in binge eating are distinct—despite being similar—constructs. Despite the overlap related to binge eating behaviors, the MEC10 captures only a part of the FA behaviors specific to binge eating, which is not exhaustive of all the facets of FA (e.g., craving, withdrawal, tolerance). Therefore, the latent correlation between the mYFAS2.0 and the MEC10 is hypothesized to be moderate to high but not excessively high, supporting their discriminant validity.

## 2. Materials and Methods

A cross-sectional study design was used. Participants were consecutively enrolled at the IRCCS Istituto Auxologico Italiano, Ospedale San Giuseppe, Verbania, Italy, among inpatients during the first week of residential rehabilitation treatment for weight reduction lasting up to one month. The inclusion criteria were as follows: being over 18 years old, having a body mass index (BMI) above 35, and being a native Italian speaker. Exclusion criteria were as follows: inability to complete the assessment due to issues related to perception, cognition, etc., and failure to provide informed consent to participate in the study. Data from this study are part of a larger research project [28,41].

### 2.1. Measures

The participants underwent a clinical interview conducted by a psychologist to determine their eligibility for the study. In addition, a self-report survey that included the following questionnaires was administered together with a ‘Socio-demographic and medical sheet’ providing information such as age, biological sex, weight, height, and BMI.

The Measure of Eating Compulsivity—Italian Version (MEC10-IT) [27,28] is a self-report questionnaire that evaluates compulsive eating within the broader framework of FA. The MEC10-IT comprises 10 items, each scored on a 5-point Likert-type scale with partial semantic autonomy, ranging from 0 = ‘Very Untrue’ to 4 = ‘Very True’. It has a unidimensional structure, and the total score is obtained by summing the items’ scores. Higher scores correspond to higher levels of eating compulsivity. The MEC10 has demonstrated good reliability and psychometric properties [28]. In this study, its internal consistency was evaluated using McDonald’s ω (ω total = 0.95), Cronbach’s α (α = 0.94), and the greatest lower bound (glb = 0.96), all of which demonstrated adequacy.

The Binge Eating Scale (BES) [42] is a self-report questionnaire to assess the severity of binge eating in both the general [9] and clinical populations [21]. It consists of 16 items divided into two subscales: the Behaviors subscale (B) consists of 8 items describing behavioral expressions of BED (e.g., consuming large amounts of food and/or eating fast), while the Feelings/Cognitions (FC) subscale includes another 8 items evaluating the feelings and cognitive thoughts associated with compulsive eating (i.e., thinking excessively about food). Each item has from 3 to 4 different alternative responses, consisting of statements with partial semantic independence, which are ordered by increasing severity. A numerical value is assigned to each statement chosen by the respondent (ranging from ‘no severity’ = 0 to ‘severe’ = 3), reflecting the severity of symptoms of BED. The total scores can be computed by adding the answers to the items on the subscale of Feelings/Cognitions (FC) and the subscale of Behaviors (B), and also a general total score for all items combined [21]. The general total score of the BES ranges from 0 to 46, with values below 17 indicating minimal problems with binge eating, values between 18 and 26 suggesting moderate binge eating, and values above 27 suggesting severe binge eating [43]. Several studies have supported the reliability and validity of the BES as an assessment tool for eating-related pathologies [42,44,45]. Moreover, recent evidence from samples with obesity has shown that the BES has a high sensitivity and specificity in distinguishing between compulsive eaters and non-compulsive (‘normal’) eaters [46,47]. In this study, the BES showed adequate internal consistency, measured through McDonald’s ω (BES total scale = 0.90, FC scale = 0.85, and B scale = 0.86), Cronbach’s α for comparability with other studies (BES total scale = 0.89, FC scale = 0.81, and B scale = 0.82), and glb (BES total scale = 0.92, FC scale = 0.84, and B scale = 0.86).

The Modified Yale Food Addiction Scale 2.0 (mYFAS2.0) [24,48] is a self-report questionnaire measuring the presence and frequency of addictive eating behaviors. It consists of 13 items scored on an eight-point Likert-type scale, ranging from 0 (=‘never’) to 7 (=‘every day’), to assess the frequency of these behaviors. Eleven of these items assess the diagnostic criteria of the DSM-5 for substance use disorders (SUD) [26], while the remaining two items evaluate food-related impairment and the distress perceived over the past year. Successively, thresholds were determined [24,25], allowing each item to be re-scored dichotomously as either ‘met the criterion’ (=1) or ‘did not meet the criterion’ (=0). This enables the computation of two different scores as follows: (a) the symptom count, which represents the sum of the diagnostic criteria met and ranges from 0 to 11; and (b) the diagnostic score, which requires the presence of criteria for impairment and distress. To formulate a diagnosis of FA, both the symptoms count and distress/impairment score are needed, resulting in different combinations as follows: mild FA = 2–3 symptoms plus impairment or distress; moderate FA = 4–5 symptoms plus impairment or distress; severe FA = 6 or more symptoms plus impairment or distress [49]. The mYFAS2.0 showed good internal consistency values in this study (ω total = 0.87, KR-20 coefficient = 0.84, and glb = 0.88).

### 2.2. Data Analysis Strategy

The statistical analyses were conducted using the R software (version 4.2.2) [50] and the R Studio environment [51]. Several R packages were utilized for the analyses, including psych [52], dplyr [53], reshape [54], ggplot2 [55], arsenal [56], lavaan [57], semTools [58], semPlot [59], and likert [60].

#### 2.2.1. Preliminary Analysis

Descriptive statistics were used to describe the socio-demographic and physical characteristics of the sample. Additionally, they were employed to examine the distributions of the items and the levels of the variables.

The item correlations were explored using the Spearman correlation coefficient.

The internal consistency was evaluated for Likert-type scales with McDonald’s ω (suitable also for non-τ-equivalent models). Cronbach’s α was also reported for comparability with other studies despite its tendency to underestimate reliability [61,62]. For scales with a dichotomous response scale, the KR-20 coefficient was also reported. Additionally, the greatest lower bound (glb) was reported [63,64].

#### 2.2.2. Discriminant Validity

Discriminant validity was evaluated according to recent methodological guidelines [33]. A structural equation model (SEM) was specified to include the measurement models of the three scales under consideration, with items loading on their respective latent factors. Scaling on the latent variable was applied, and the estimator used was diagonally weighted least squares (DWLS), chosen for its suitability for categorical and dichotomous items [65,66,67,68,69]. The latent correlations among the factors were freely estimated, along with 95% confidence intervals (95% CI).

The goodness-of-fit of the SEM to the data was evaluated according to the following guidelines: a non-statistically significant chi-square statistic (χ^2^), a comparative fit index (CFI) > 0.95 for good fit, robust root mean square error of approximation (RMSEA) with the 90% confidence interval (CI) of RMSEA < 0.08, and a standardized root mean residual (SRMR) < 0.08 [65,66,67,69].

According to the methodological guidelines based on simulation studies, the presence of discriminant validity was evaluated using two techniques.

Among the correlation-related techniques, two techniques represent a powerful way to establish the presence or absence of discriminant validity: ρxx(cut) and CIxx(cut)—the former tests whether the point estimate of their latent correlation (ρxx) for every pair of latent factors falls below a certain cutoff. Conversely, CIxx(cut) tests whether the 95% confidence interval (CI) of the correlation between each pair of latent factors is below a certain cutoff point. Discriminant validity between two scales is only supported if the upper bound of the 95% CI of the correlations between the latent factors does not exceed the threshold of 0.85 [33].

Among the techniques focusing on model fitting, we also considered χ^2^(cut) and CFI(cut). First, two models have to be fitted, and then their fits are compared as follows: the unconstrained model, with the freely estimated latent factors’ correlation, and a second constrained model, where the latent correlation is fixed to a cutoff value. Second, the fit of the two models (unconstrained and constrained) is compared and evaluated using two criteria, χ^2^(cut) and CFI(cut). Specifically, regarding χ^2^(cut), if the unconstrained model with freely estimated correlation (showing a value < 0.85) fits better than the model with the fixed correlation (at 0.85), it means that there are no problems with discriminant validity. Similarly, if the unconstrained model with freely estimated correlation (reporting a value > 0.85) fits better than the model with constrained correlation (at 0.85), it means that there are problems with discriminant validity. If the difference in χ^2^(cut) between the two models (unconstrained vs. constrained) is not statistically different from zero, it means that there are problems of discriminant validity.

The CFI(cut) consists of comparing the ΔCFI between the constrained and unconstrained models against the 0.002 cutoff. A ΔCFI above the 0.002 cutoff in favor of the model with the lower latent correlation supports discriminant validity, while a ΔCFI below the 0.002 cutoff indicates the absence of discriminant validity because the ‘real’ correlation is not lower than 0.85.

### 2.3. Sample Size Determination

Given the purpose of the present investigation, the chosen measurement tools, and the data analysis strategy, the sample size was determined a priori according to the ‘n: q criterion’. Here, ‘n’ represents the number of statistical units (participants), while ‘q’ represents the number of free parameters to be estimated in the model [70,71]. To guarantee a ratio of 5 statistical observations for each model parameter (with the DWLS estimator, where the number of free parameters was 138), the minimum required sample size to reach was calculated as 5 × 138 = 690.

## 3. Results

### 3.1. Preliminary Analysis

#### 3.1.1. Participants

The sample consisted of 717 adult clinical patients with severe obesity (BMI > 35, mean 43.284 ± 6.962) seeking weight reduction treatment. Their mean age was 53.681 years (±12.742), ranging from 18 to 87 years old. The sample was balanced in terms of biological sex, comprising 403 women (56.21%) and 314 men (43.79%). Descriptive statistics of socio-demographic and physical characteristics, as well as variable levels, are reported in Table 1, which stratifies the sample by biological sex.

#### 3.1.2. Item Properties

**Figure 1:**
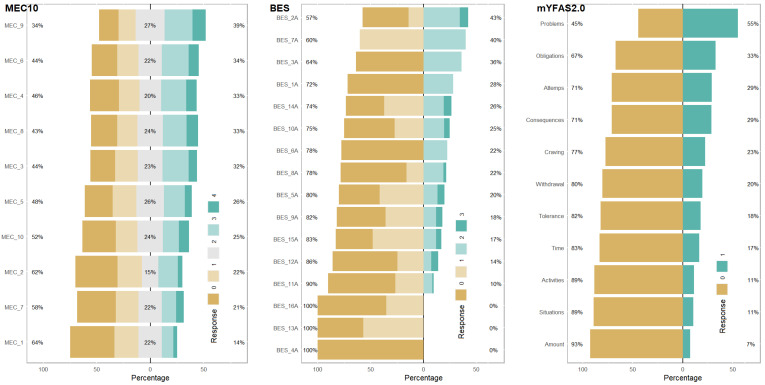
Distribution of the item response categories and their percentages.

The descriptive statistics of the items are reported in Table 2, showing that the skewness and kurtosis values were generally within the recommended ranges, suggesting that most items can be considered to be normally distributed. Figure 1 illustrates the distribution of response categories to the items and their percentages. Given that these screening questionnaires are related to pathological behaviors and feelings associated with eating disorders, certain categories are endorsed more frequently than others.

**Figure 2:**
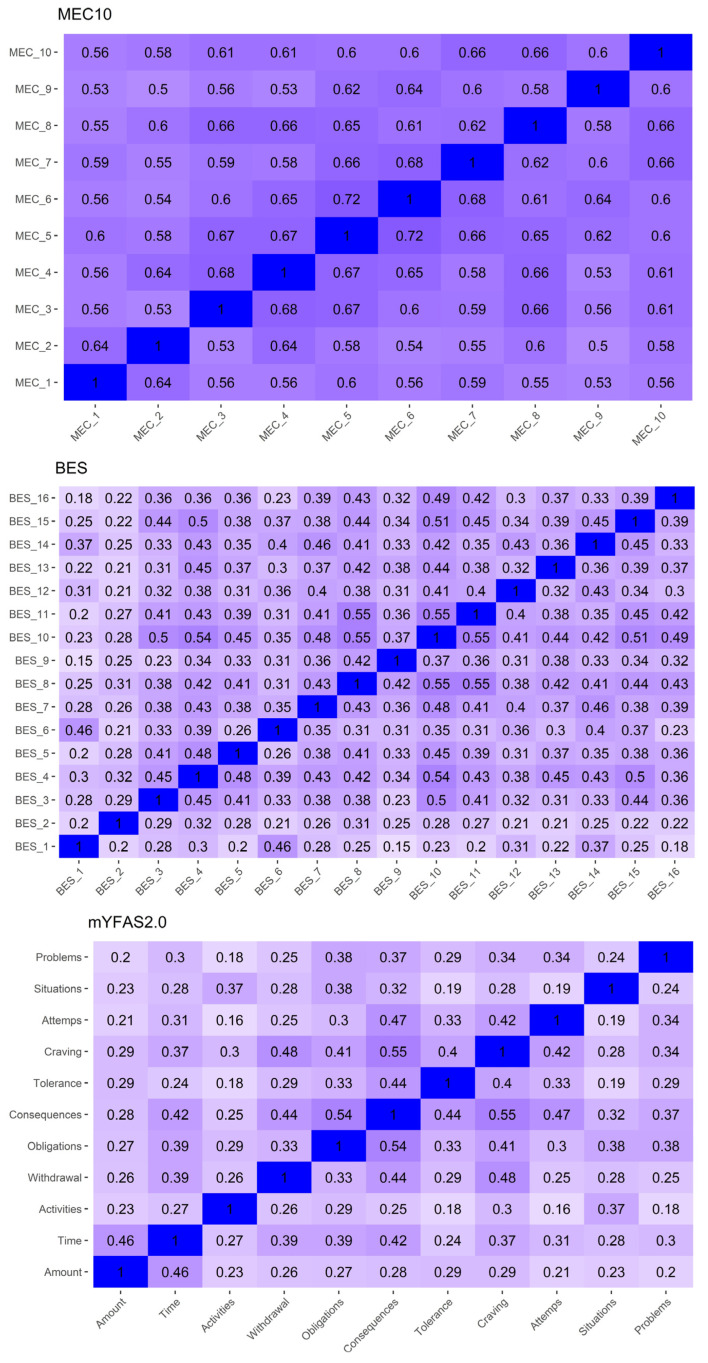
Heatmap of the correlations among items of the Measure of Eating Compulsivity 10 (MEC10), Modified Yale Food Addiction Scale 2.0 (mYFAS2.0), and Binge Eating Scale (BES). Note: The intensity of the color indicates the strength of the correlation.

Figure 2 displays the Spearman correlations between the items on each scale. The intensity of the color indicates the strength of the correlation. All items within the same scale were positively associated (*p* < 0.05).

### 3.2. Assessing Discriminant Validity through SEM

First, correlation-related techniques were employed, including the point estimate of the latent correlation and its 95% CI, which should not exceed the threshold. Second, model-fit techniques were utilized, such as Δχ^2^(cut) and ΔCFI(cut).

#### 3.2.1. Unconstrained Model: ρxx(Cut) and CIxx(Cut)

**Figure 3:**
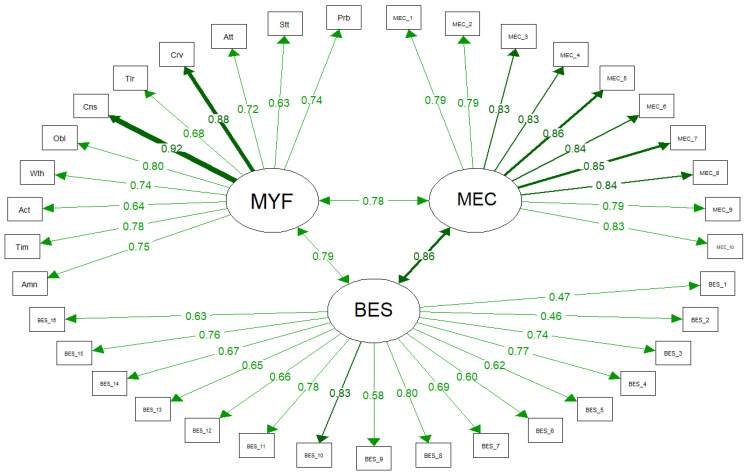
Plot of the structural equation model assessing discriminant validity. Note: MYF = mYFAS2.0; MEC10 = Measure of Eating Compulsivity 10; BES = Binge Eating Scale.

Figure 3 depicts the graphical representation of the SEM, illustrating the three measurement models of the following measures: MEC10, BES, and mYFAS2.0. The arrows between the latent factors (represented within the circles) signify the latent factors themselves. Items are represented within rectangles, with arrows extending from the factors (circles) to items indicating the item loadings. Each path is labeled to indicate its standardized estimate, and the thickness of the paths reflects their strength.

The SEM model, incorporating the measurement models of the three considered measures, showed an excellent fit to the data, as indicated by the following fit indices. The χ^2^ _(df = 626)_ = 940.048 was associated with a statistically significant *p*-value of <0.001, primarily due to the large sample size. The CFI (=0.998) suggests an excellent fit, along with the RMSEA (=0.026), which, with a 90% CI of RMSEA [0.023, 0.030], indicated a high probability (equal to 1) of the RMSEA being below 0.050. Finally, the SRMR was 0.048.

Examination of the item loadings onto their latent factor revealed that they were all highly associated with the measured construct and in a statistically significant manner (*p* < 0.001), thereby supporting the scale dimensionality and construct validity as being in line with the previous validations. Table 3 shows the item loadings on their respective latent factors, along with their explained variance (r^2^).

Table 4 displays the three latent correlations between the three factors and their 95% CI in the unconstrained model. All three latent factors exhibited strong and statistically significant positive correlations. In detail, the mYFAS2.0, considered the gold standard assessment tool for FA, showed strong and comparable correlations with both MEC10 (0.783) and BES (0.786).

##### MEC10 and BES

The latent correlation between MEC10 and BES had a standardized point estimate of 0.856, with a 95% CI ranging from the lower bound of 0.844 to the upper bound of 0.867. Two of these three values exceed the recommended threshold for latent factor correlations (values > 0.850), which should not be surpassed to support the discriminant validity of the two measures. Indeed, a correlation that is ‘too’ high between the two measures indicates the absence of discriminant validity and suggests potential overlap between these questionnaires.

##### mYFAS2.0 and MEC10

The latent correlation between MEC10 and mYFAS2.0 had a standardized point estimate of 0.783, with a 95% CI ranging from the lower bound of 0.766 to the upper bound of 0.799. Since all these values are below the recommended threshold for latent factor correlations (values < 0.850), the not ‘too’ high correlation between MEC10 and mYFAS2.0 indicates the presence of discriminant validity between them.

##### mYFAS2.0 and BES

The latent correlation between the latent factors of BES and mYFAS2.0 had a standardized estimate of 0.786 with a 95% CI [0.768, 0.804]. Since these values fall below the threshold of.850, discriminant validity between BES and YFAS is supported.

In summary, based on the point estimate of the latent correlations and their 95% CIs, the discriminant validity of mYFAS2.0 is supported both with MEC10 and BES. In contrast, discriminant validity between MEC10 and BES is absent.

#### 3.2.2. Comparison of the Unconstrained and Constrained Models: χ^2^(cut) and CFI(cut)

According to the guidelines, also the χ^2^(cut) and CFI(cut) techniques were used to test discriminant validity. Specifically, the χ^2^ fit of the previous unconstrained model (allowing free estimation of all latent correlations) was compared to another model in which the latent correlation between the constructs (MEC10 and BES or MEC and mYFAS2.0) was constrained to a cutoff value (i.e., 0.850).

Subsequently, the model comparison relied on χ^2^(cut) and CFI(cut). Table 5 presents the fit of the model(s) and their comparison using χ^2^(cut) and CFI(cut).

##### MEC10 and BES

Considering Δχ^2^(cut), when the latent correlation between MEC10 and BES was constrained to be 0.850, the Δχ^2^(cut) was 0.992, and it was not statistically different from 0 (*p* = 0.319). Considering the reference point of 0.850, the correlation between MEC10 and BES was not significantly different (less) from 0.85, indicating a lack of discriminant validity between MEC10 and BES.

Regarding ΔCFI(cut), the results indicate that both the models had an equal CFI, resulting in a Δ of 0. This suggests the absence of discriminant validity between MEC10 and BES (CFI unconstrained = 0.998; CFI constrained at 0.85 = 0.998; and ΔCFI(cut) = 0).

Two other models were specified, constraining the latent correlation of MEC10 and BES to be equal to 0.90 and then to 0.95. The model with a latent correlation of 0.90 was invariant when compared to the unconstrained model, but the model with a latent correlation of 0.95 was not invariant compared to the unconstrained one. This means that the latent correlation between MEC10 and BES is significantly less than 0.95 but not significantly less than 0.90 or 0.85—supporting the absence of discriminant validity between these measures.

##### mYFAS2.0 and MEC10

The same approach was used to test the discriminant validity of MEC10 and mYFAS2.0 through Δχ^2^(cut) and ΔCFI(cut). A model with a latent correlation between MEC10 and mYFAS2.0 constrained to 0.85 was compared to the unconstrained model.

Δχ^2^(cut) showed a Δχ^2^ of 59.745 that was statistically significant (*p* < 0.001), different than the unconstrained model, supporting the discriminant validity of MEC10 and mYFAS2.0. ΔCFI(cut) showed that the ΔCFI between the unconstrained model and the model with the latent correlation constrained to 0.85 was only −0.001 below the threshold of 0.002, but this suggests that the difference in goodness-of-fit between the models is minimal, and ΔCFI(cut) may be too conservative.

##### mYFAS2.0 and BES

For completeness, the discriminant validity of mYFAS2.0 and BES was also tested through a model-fit comparison, with their latent correlation fixed at 0.850. The Δχ^2^(cut) between the constrained and unconstrained models was 46.325 and statistically significant, supporting discriminant validity. The ΔCFI(cut) was 0.001, below the threshold, but reflecting the good fit of both models and potentially being too conservative.

## 4. Discussions

The present research was the first aimed at assessing the discriminant validity of two measures of food addiction (FA), MEC10 and mYFAS2.0, in a large sample of inpatients with severe obesity (BMI > 35). A SEM approach was used in accordance with the recent methodological guidelines within the field of test validity. A combination of psychometric techniques was used to assess the presence or absence of discriminant validity between measures as follows: (i) In an unconstrained model, the latent variable correlation and its 95% CI were estimated as free parameters—their values should be below the threshold of 0.85 to support discriminant validity; (ii) the Δχ^2^ and ΔCFI were evaluated between the unconstrained model and a second model in which each target latent correlation was constrained to be fixed at 0.85—a significantly better fit of the unconstrained model supported discriminant validity. These steps were repeated for each of the three latent correlations.

The results showed that the discriminant validity of mYFAS2.0, the gold standard measure of FA, was supported both with MEC10 and BES. This means that mYFAS2.0 measures a construct distinct from those assessed by MEC10 and BES. In contrast, the data did not support discriminant validity between MEC10 and BES. In this regard, the results from the point estimate, 95% CI, Δχ^2^, and ΔCFI were consistent.

The presence of discriminant validity between mYFAS2.0 and both MEC10 and BES is theoretically expected because mYFAS2.0 specifically assesses FA in its behavioral and substance use components, whereas MEC10 and BES ‘only’ capture binge eating behaviors that are strongly related to FA but distinct from it. Indeed, BED is not always associated with FA and vice versa.

Differently, the absence of discriminant validity between MEC10 and BES suggests that MEC10 does not measure FA as originally intended but rather could be a measure of binge eating. Thus, MEC10 and BES seem to measure the same construct, and they are equally related to FA, as measured by mYFAS2.0. Therefore, MEC10 represents an optimal measure of binge eating but not of FA.

The findings of the present research study are consistent with the current literature and previous scientific evidence [28], which indicate that MEC10 is more similar to BES than to mYFAS2.0.

### 4.1. FA and BED: Constructs Differences

Highlighting the difference between the constructs of FA and BED is crucial to grasp the importance of these findings. The hallmark of BED is the episodes of binge eating, characterized by the intake of an unusually high quantity of food in a short time [72]. However, FA may not necessarily manifest with binge eating episodes, and binge eating episodes may not necessarily exhibit specific characteristics of FA, such as craving, tolerance, or withdrawal, which are typical of addictions.

MEC10 captures only one aspect of FA, which is eating compulsivity manifested as a binge eating episode. Thus, MEC10 does not capture all the other aspects of FA. Differently, mYFAS2.0 provides a broader reflection of the FA constructs, incorporating all relevant facets.

In summary, patients with BED are highly likely to also have FA, but those with FA may not necessarily have BED because FA encompasses various aspects (11 criteria), including but not limited to episodes of binge eating.

### 4.2. MEC10 or BES?

As the discriminant validity of MEC10 and BES is not supported, and both assess binge eating, one may wonder which measure to use for assessing binge eating behaviors and their correlates. The answer depends on various considerations, such as the characteristics of the respondents (level of psychological and cognitive functioning), the context of the application (research or clinical), and the type of study (quantitative vs. qualitative). However, using both in clinical or research studies will result in redundancy, higher resource consumption, longer assessment time, overburden for respondents, and reduced attention, consequently leading to lower accuracy in answers to long assessment batteries. In fact, the literature highlights how assessment tools in health contexts should be short, acceptable for respondents, and feasible for researchers and/or clinicians [73].

In terms of the cognitive load required of the respondents, MEC10 and BES consistently differ. MEC10 has a lean structure consisting of 10 Likert-type items and excellent psychometric properties. The BES also exhibits optimal psychometric properties, but its structure is more complex. It comprises 16 groups of items, each containing four different sentences with varying severity levels (partial semantic autonomy). Consequently, the respondents have to read and elaborate information from 16 × 4 sentences, resulting in a high cognitive load and consistently extending the assessment time. Moreover, from a psychometric perspective, the response alternatives in the BES are not consistent across all item groups: while 14 out of 16 items have four response alternatives, only 2 out of 16 items have three response options. This discrepancy represents a problem in SEM and measurement models. Regarding the content’s validity, the longer form of the BES can capture more facets of the constructs, albeit at the cost of a considerable cognitive load. For these reasons, MEC10 should be preferred for the assessment of binge eating due to its fewer items and maintained high accuracy.

### 4.3. Limitations

The sample consisted solely of adult inpatients with severe obesity, and this may restrict the generalizability of the results. Despite the large sample size, the sample only partially represented the clinical population because it was not balanced, as patients with obesity were included, but there were no patients with a diagnosis of BED. Moreover, the sample was unbalanced in terms of obesity classes, with Class 2 being expected to be more prevalent than Class 3. Additionally, a limited number of self-report assessment tools were used, such as the MEC10, BES, and mYFAS2.0. It is worth noting that self-reports have some inherent limitations, such as a susceptibility to social desirability bias and the potential hiding of different symptom configurations behind the same score [74]. Moreover, the cross-sectional research design did not allow for observation of whether these findings remained consistent over time. Finally, in this study, differences associated with biological sex and gender were not considered [75], nor were factors related to broader social [76,77] and environmental conditions [78]. Future studies are warranted to either confirm or refute these findings and to broaden their applicability to other populations. For example, longitudinal studies conducted across samples from various populations, such as patients with eating disorders and the general population, could delve deeper into these topics. These studies have the potential to validate the present findings and extend their relevance to diverse populations.

### 4.4. Strengths

The strengths of this present research are both clinical and methodological.

In terms of clinical strengths, this study is the first to assess the discriminant validity of measures of FA and binge eating using statistically robust methods and techniques. The findings reveal that the MEC10, previously improperly considered a measure of FA, is more likely a measure of binge eating, as indicated by its lack of discriminant validity with the BES.

From a methodological perspective, the focus on discriminant validity is a key strength, as it is an important property of an assessment tool and a relevant facet when evaluating construct validity [79]. In the present research, the statistical analyses relied on robust and sound methodologies such as SEM, which allowed for the precise estimation of latent factors and their correlations by minimizing measurement error variability. From an applicative perspective, this research provides an example of the assessment of discriminant validity in health psychology through SEM. Indeed, when assessing discriminant validity, it is common practice to evaluate observed correlations between scales, which may include measurement error. However, latent correlations between factors are frequently neglected in scale validation [33]. Regarding the methodology, a large clinical sample of adult inpatients with obesity who received a formal diagnosis from physicians and psychologists was used. This ensured accurate estimates, and the sample was well-balanced in terms of biological sex and age.

In general, considering the limitations and strengths mentioned above, these findings hold significance and novelty within the context of the current literature on FA and eating disorders. They have the potential to inform future research and clinical practice in these fields.

### 4.5. Future Research

Future studies are needed to replicate or disconfirm these findings in different populations, such as patients with BED, community samples, ‘normal’ weight individuals, and adolescents, both within Italy and across other countries. Additionally, the role of other factors, such as gender and age, warrants investigation in future research.

Furthermore, there are many scales whose discriminant validity has never been rigorously tested, as shown in this study. These steps [33] could be applied to test the discriminant validity of various measures.

Moreover, future studies may develop and test new methods to assess discriminant validity. Finally, future research in statistics and psychometrics may aim to unravel the current ambiguities surrounding discriminant validity, particularly regarding its teaching and application in clinical and research contexts.

### 4.6. Further Methodological Considerations

If discriminant validity is defined as the extent to which two measures of similar but distinct constructs are associated but not with a too high association, the question arises: how low is low enough to consider them distinct? There is an ongoing debate regarding the use of thresholds versus considering a continuum and the subsequent interpretation. For instance, the 0.85 threshold has been considered the optimal cutoff point based on methodological literature and simulation studies; however, its validity may be questionable. Some researchers may consider it problematic to determine whether two scales measure different constructs solely based on a cutoff value. While rules of thumb and cutoffs can be useful, exercising good judgment is always necessary in their interpretation. For example, biological sex and gender are highly correlated variables (0.995) [26], yet it is undeniable that they refer to distinct constructs. As noted by Rönkkö & Cho [33] on page 12, thresholds have to be interpreted: “the existence of a threshold, that is, a correlation below a certain level has no problem with discriminant validity but does not dictate a specific cutoff, thus also allowing the value of the correlation to be interpreted instead of simply tested”.

Testing discriminant validity is important for at least two reasons. From the psychometric perspective, not testing the discriminant validity of a scale may result in the use of scales that are overly correlated, leading to multicollinearity issues in linear models. From a pragmatic standpoint, choosing a scale without considering its discriminant validity with similar scales could lead to construct redundancy and unnecessary prolongation of assessment times, ultimately reducing accuracy.

Discriminant validity is a facet of validity that is often neglected. Additionally, ambiguity and confusion surrounding discriminant validity may arise from poor theoretical clarity and conceptual confusion. At times, discriminant validity is mistakenly equated with the test’s ability to distinguish between different groups (known group validity), such as clinical versus nonclinical populations, or confused with criterion validity (the ability to predict criteria associated with the measured construct) or divergent validity (e.g., negative or low correlations).

Interestingly, the simulation studies [33] on the present methodology were from the field of organizational settings and management and were then applied in the psychological field with encouraging results. Such different disciplines rely on a common ground of methodology, statistical analyses, techniques, and measurement test validity.

Psychometrically sound measures represent a crucial resource for psychological assessment to outline face-to-face and digital interventions, especially when dealing with patients with medical conditions such as obesity or respiratory and cardiovascular diseases [80,81,82,83,84].

The scientific literature highlights that it is of crucial importance to assess the discriminant validity of measurement scales before testing research hypotheses and investigating relationships between constructs [85].

## 5. Conclusions

In conclusion, the discriminant validity between MEC10 and BES is absent (ρ point estimate = 0.856, 95% CI [0.844, 0.867]); therefore, MEC10 is a promising questionnaire with excellent psychometric properties but should be considered as a measure of BED and not FA. These findings support the validity and suitability of MEC10, a psychometrically sound tool that is useful for measuring binge eating behaviors associated with FA, both for research and clinical purposes. On the contrary, the discriminant validity of mYFAS2.0 and MEC10 is supported (ρ point estimate = 0.783, 95% CI [0.766, 0.799]), as they measure similar but distinct constructs that are not too highly correlated.

The SEM methodology with latent variables allowed the investigation of the psychometric properties and construct validity of these psychological assessment tools, focusing in particular on discriminant validity.

## Figures and Tables

**Table 1:** Descriptive statistics of the sample socio-demographic, physical, and psychological variables.

	Males (*n* 314)	Females (*n* 403)	Total (*n* = 717)
**Age**			
Mean (SD)	54.557 (12.401)	52.924 (13.138)	53.628 (12.842)
Range	18–87	18–80	18–87
**Weight in kg**			
Mean (SD)	130.872 (24.523)	110.288 (19.471)	118.876 (23.963)
Range	82.600–270	70–220.200	70–270
**Height in meters**			
Mean (SD)	1.736 (0.074)	1.594 (0.075)	1.653 (0.102)
Range	1.500–1.950	1.400–1.830	1.400–1.950
**Body Mass Index**			
Mean (SD)	43.345 (7.132)	43.332 (6.395)	43.337 (6.707)
Range	35.077–86.182	35.156–83.210	35.077–86.182
**MEC10**			
Mean (SD)	14.571 (9.834)	16.649 (10.595)	15.745 (10.315)
Range	0–37	0–40	0–40
**BES**			
Mean (SD)	25.667 (7.626)	28.536 (8.849)	27.287 (8.454)
Range	15–52	15–57	15–57
**mYFAS2.0**			
Mean (SD)	2.231 (2.449)	2.756 (2.889)	2.527 (2.717)
Range	0–11	0–11	0–11
***n* (%)**	Males (*n* 314)	Females (*n* 403)	Total (*n* = 717)
**FA diagnosis**			
No FA	241 (77.2%)	287 (70.9%)	528 (73.6%)
FA	71 (22.8%)	118 (29.1%)	189 (26.4%)
**Severity of FA diagnosis**			
Mild FA	17 (23.9%)	21 (17.8%)	38 (20.1%)
Moderate FA	22 (31.0%)	30 (25.4%)	52 (27.5%)
Severe FA	32 (45.1%)	67 (56.8%)	99 (52.4%)
**Only FA diagnosis**			
Not only FA	276 (88.5%)	369 (91.1%)	645 (90.0%)
Only FA	36 (11.5%)	36 (8.9%)	72 (10.0%)
**BED diagnosis**			
No BED	248 (79.5%)	280 (69.1%)	528 (73.6%)
BED	64 (20.5%)	125 (30.9%)	189 (26.4%)
**Only BED diagnosis**			
Not only BED	283 (90.7%)	362 (89.4%)	645 (90.0%)
Only BED	29 (9.3%)	43 (10.6%)	72 (10.0%)

Note: MEC10 = Measure of Eating Compulsivity 10; mYFAS2.0 = symptom count of the Modified Yale Food Addiction Scale 2.0; BES = Binge Eating Scale. FA = food addiction; BED = binge eating disorder.

**Table 2:** Descriptive statistics of the items of the MEC10, BES, and mYFAS2.0.

MEC10	Mean	sd	Median	Min	Max	Range	Skew	Kurtosis
MEC_1	1.126	1.172	1	0	4	4	0.698	−0.562
MEC_2	1.250	1.260	1	0	4	4	0.561	−0.995
MEC_3	1.725	1.278	2	0	4	4	0.081	−1.163
MEC_4	1.700	1.349	2	0	4	4	0.135	−1.262
MEC_5	1.582	1.244	2	0	4	4	0.220	−1.042
MEC_6	1.766	1.313	2	0	4	4	0.060	−1.212
MEC_7	1.344	1.289	1	0	4	4	0.544	−0.862
MEC_8	1.756	1.328	2	0	4	4	0.099	−1.183
MEC_9	1.985	1.286	2	0	4	4	−0.137	−1.059
MEC_10	1.512	1.322	1	0	4	4	0.380	−1.024
**BES**	**mean**	**sd**	**median**	**min**	**max**	**range**	**skew**	**kurtosis**
BES_1	2.384	0.987	2	1	4	3	0.020	−1.068
BES_2	2.071	1.049	2	1	4	3	0.279	−1.388
BES_3	1.639	0.936	1	1	4	3	1.416	0.953
BES_4	2.059	0.936	2	1	4	3	0.587	−0.523
BES_5	1.877	0.877	2	1	4	3	0.822	−0.008
BES_6	1.722	0.683	2	1	3	2	0.413	−0.849
BES_7	1.636	0.940	1	1	4	3	1.385	0.815
BES_8	1.619	0.884	1	1	4	3	1.062	−0.251
BES_9	1.773	0.876	2	1	4	3	0.979	0.187
BES_10	1.827	0.928	2	1	4	3	0.757	−0.565
BES_11	1.473	0.704	1	1	4	3	1.368	1.185
BES_12	1.589	0.883	1	1	4	3	1.459	1.203
BES_13	1.778	0.980	1	1	4	3	1.121	0.158
BES_14	1.971	0.919	2	1	4	3	0.608	−0.546
BES_15	1.870	0.809	2	1	4	3	0.808	0.332
BES_16	1.749	0.768	2	1	4	3	0.497	−1.033
**mYFAS2.0**	**mean**	**sd**	**median**	**min**	**max**	**range**	**skew**	**kurtosis**
Amount	0.074	0.262	0	0	1	1	3.250	8.576
Time	0.167	0.374	0	0	1	1	1.778	1.164
Activities	0.114	0.318	0	0	1	1	2.418	3.854
Withdrawal	0.197	0.398	0	0	1	1	1.523	0.321
Obligations	0.329	0.470	0	0	1	1	0.726	−1.475
Consequences	0.289	0.453	0	0	1	1	0.931	−1.136
Tolerance	0.179	0.383	0	0	1	1	1.675	0.808
Craving	0.226	0.418	0	0	1	1	1.308	−0.290
Attempts	0.291	0.455	0	0	1	1	0.916	−1.163
Situations	0.107	0.310	0	0	1	1	2.531	4.411
Problems	0.554	0.497	1	0	1	1	−0.216	−1.956

Note: MEC10 = Measure of Eating Compulsivity 10; mYFAS2.0 = symptom count of the Modified Yale Food Addiction Scale 2.0; BES = Binge Eating Scale.

**Table 3:** Factor loadings of the items on latent factors in the SEM model, as represented in Figure 3.

MEC10 Loadings					BES Loadings					mYFAS2.0 Loadings				
Item	MEC10	BES	mYFAS2.0	r^2^	Item	MEC10	BES	mYFAS2.0	r^2^	Item	MEC10	BES	mYFAS2.0	r^2^
MEC_1	0.793	0	0	0.629	BES_1	0	0.465	0	0.217	Amount	0	0	0.752	0.566
MEC_2	0.789	0	0	0.622	BES_2	0	0.460	0	0.211	Time	0	0	0.780	0.608
MEC_3	0.831	0	0	0.690	BES_3	0	0.741	0	0.549	Activities	0	0	0.642	0.412
MEC_4	0.833	0	0	0.694	BES_4	0	0.772	0	0.596	Withdrawal	0	0	0.740	0.547
MEC_5	0.855	0	0	0.731	BES_5	0	0.624	0	0.390	Obligations	0	0	0.802	0.644
MEC_6	0.838	0	0	0.703	BES_6	0	0.602	0	0.363	Consequences	0	0	0.917	0.842
MEC_7	0.853	0	0	0.728	BES_7	0	0.686	0	0.471	Tolerance	0	0	0.685	0.469
MEC_8	0.836	0	0	0.698	BES_8	0	0.803	0	0.644	Craving	0	0	0.881	0.777
MEC_9	0.787	0	0	0.620	BES_9	0	0.579	0	0.335	Attempts	0	0	0.721	0.520
MEC_10	0.829	0	0	0.687	BES_10	0	0.834	0	0.696	Situations	0	0	0.633	0.400
					BES_11	0	0.782	0	0.612	Problems	0	0	0.739	0.546
					BES_12	0	0.657	0	0.432					
					BES_13	0	0.652	0	0.426					
					BES_14	0	0.671	0	0.450					
					BES_15	0	0.758	0	0.575					
					BES_16	0	0.630	0	0.396					

Note: MEC10 = Measure of Eating Compulsivity 10; mYFAS2.0 = Modified Yale Food Addiction Scale 2.0; BES = Binge Eating Scale. r^2^ = r squared.

**Table 4:** Covariances among latent factors and 95% confidence intervals in the unconstrained model.

Unconstrained Model						
	Point Std. Estimate	95% CI				
Latent Factors		Lower	Upper	Std. Err.	z-Value	*p*-Value
MEC10 ~~	-	-	-	-	-	-
mYFAS2.0	0.783	0.766	0.799	0.008	93.394	<0.001
BES	0.856	0.844	0.867	0.006	148.023	<0.001
BES ~~	-	-	-	-	-	-
mYFAS2.0	0.786	0.768	0.804	0.009	85.595	<0.001

Note: MEC10 = Measure of Eating Compulsivity 10; mYFAS2.0 = Modified Yale Food Addiction Scale 2.0; BES = Binge Eating Scale.

**Table 5:** Assessment of discriminant validity. Fit of models (on the left) and model comparison with the unconstrained model as a reference (on the right). The upper part of the table pertains to the correlation between MEC10 and BES. The middle part concerns the correlation between MEC10 and mYFAS2.0. The lower part concerns the correlation between mYFAS2.0 and BES.

**MEC10 ~~ BES** **Point est. 0.856** **95% CI [0.844, 0.867]**	**Model fit**						**Model comparison**					
	**X^2^**	**df**	***p***	**CFI**	**RMSEA**	**SRMR**	**ΔX^2^**	**Δdf**	***p***	**ΔCFI**	**ΔRMSEA**	**DV**
Unconstrained model	940.048	626	<0.001	0.998	0.026	0.048	-	-	-	-	-	-
Constrained at 0.85	941.040	627	<0.001	0.998	0.026	0.048	0.992	1	0.319	0	0	No
Constrained at 0.90	996.47	627	<0.001	0.997	0.029	0.049	56.425	1	<0.001	−0.001	0.003	-
Constrained at 0.95	1184.57	627	<0.001	0.996	0.035	0.051	244.53	1	<0.001	−0.002	0.090	-
**mYFAS2.0 ~~ MEC10** **point est. 0.783** **95% CI [0.766, 0.799]**	**Model fit**						**Model comparison**					
	**X^2^**	**df**	***p***	**CFI**	**RMSEA**	**SRMR**	**ΔX^2^**	**Δdf**	***p***	**ΔCFI**	**ΔRMSEA**	**DV**
Unconstrained model	940.048	626	<0.001	0.998	0.026	0.048	-	-	-	-	-	-
Model constr. 0.85	999.793	627	<0.001	0.997	0.029	0.050	59.745	1	<0.001	−0.001	0.003	Yes
**mYFAS2.0 ~~ BES** **point est. 0.786** **95% CI [0.768, 0.804]**	**Model fit**						**Model comparison**					
	**X^2^**	**df**	***p***	**CFI**	**RMSEA**	**SRMR**	**ΔX^2^**	**Δdf**	***p***	**ΔCFI**	**ΔRMSEA**	**DV**
Model unconstrained	940.048	626	<0.001	0.998	0.026	0.048	-	-	-	-	-	-
Constrained at 0.85	986.373	627	<0.001	0.997	0.028	0.049	46.325	1	<0.001	−0.001	0.002	Yes

Note: MEC10 = Measure of Eating Compulsivity 10; BES = Binge Eating Scale; mYFAS2.0 = modified Yale Food Addiction Scale. **~~ =** latent correlation; point est. = point estimate of the correlation in the unconstrained model; 95% CI = confidence interval at 95% in the unconstrained model. X^2^ = chi-square statistic; df = degrees of freedom; *p* = *p*-value; CFI = comparative fit index; RMSEA = root means square error of approximation; Δ = difference; DV = discriminant validity.

## Data Availability

The data presented in this study are not publicly available due to privacy restrictions. Data are available on request from the corresponding author.

## References

[1] Popkin B.M., Adair L.S., Ng S.W. (2012). Global Nutrition Transition and the Pandemic of Obesity in Developing Countries. Nutr. Rev..

[2] GBD 2015 Obesity Collaborators (2017). Health Effects of Overweight and Obesity in 195 Countries over 25 Years. N. Engl. J. Med..

[3] Davis C., Curtis C., Levitan R.D., Carter J.C., Kaplan A.S., Kennedy J.L. (2011). Evidence That ‘Food Addiction’ Is a Valid Phenotype of Obesity. Appetite.

[4] Yau Y.H.C., Leeman R.F., Potenza M.N., el-Guebaly N., Carrà G., Galanter M. (2015). Biological Underpinning of Behavioural Addictions and Management Implications. Textbook of Addiction Treatment: International Perspectives.

[5] Gearhardt A.N., White M.A., Masheb R.M., Morgan P.T., Crosby R.D., Grilo C.M. (2012). An Examination of the Food Addiction Construct in Obese Patients with Binge Eating Disorder. Int. J. Eat. Disord..

[6] Linardon J., Messer M. (2019). Assessment of Food Addiction Using the Yale Food Addiction Scale 2.0 in Individuals with Binge-Eating Disorder Symptomatology: Factor Structure, Psychometric Properties, and Clinical Significance. Psychiatry Res..

[7] Manzoni G.M., Rossi A., Pietrabissa G., Mannarini S., Fabbricatore M., Imperatori C., Innamorati M., Gearhardt A.N., Castelnuovo G. (2021). Structural Validity, Measurement Invariance, Reliability and Diagnostic Accuracy of the Italian Version of the Yale Food Addiction Scale 2.0 in Patients with Severe Obesity and the General Population. Eat. Weight Disord..

[8] Murphy C.M., Stojek M.K., MacKillop J. (2014). Interrelationships among Impulsive Personality Traits, Food Addiction, and Body Mass Index. Appetite.

[9] Manzoni G.M., Rossi A., Pietrabissa G., Varallo G., Molinari E., Poggiogalle E., Donini L.M., Tarrini G., Melchionda N., Piccione C. (2018). Validation of the Italian Yale Food Addiction Scale in Postgraduate University Students. Eat. Weight Disord..

[10] Meule A., Hermann T., Kübler A. (2015). Food Addiction in Overweight and Obese Adolescents Seeking Weight-Loss Treatment. Eur. Eat. Disord. Rev..

[11] Meule A., Gearhardt A.N. (2014). Food Addiction in the Light of DSM-5. Nutrients.

[12] Pursey K.M., Stanwell P., Gearhardt A.N., Collins C.E., Burrows T.L. (2014). The Prevalence of Food Addiction as Assessed by the Yale Food Addiction Scale: A Systematic Review. Nutrients.

[13] Schulte E.M., Smeal J.K., Gearhardt A.N. (2017). Foods Are Differentially Associated with Subjective Effect Report Questions of Abuse Liability. PLoS ONE.

[14] MacLean P.S., Blundell J.E., Mennella J.A., Batterham R.L. (2017). Biological Control of Appetite: A Daunting Complexity. Obesity.

[15] Jiménez-Murcia S., Granero R., Wolz I., Baño M., Mestre-Bach G., Steward T., Agüera Z., Hinney A., Diéguez C., Casanueva F.F. (2017). Food Addiction in Gambling Disorder: Frequency and Clinical Outcomes. Front. Psychol..

[16] Rogers P.J. (2017). Food and Drug Addictions: Similarities and Differences. Pharmacol. Biochem. Behav..

[17] Brewerton T.D. (2017). Food Addiction as a Proxy for Eating Disorder and Obesity Severity, Trauma History, PTSD Symptoms, and Comorbidity. Eat. Weight Disord. Stud. Anorex. Bulim. Obes..

[18] Nolan L.J. (2017). Is It Time to Consider the “Food Use Disorder?”. Appetite.

[19] Bastianelli A., Vicentini M., Spoto A., Vidotto G. (2007). Un modello di equazioni strutturali per lo studio dei fattori di rischio nel mantenimento del disturbo da alimentazione incontrollata. G. Ital. Med. Lav. Ergon..

[20] di Giacomo E., Aliberti F., Pescatore F., Santorelli M., Pessina R., Placenti V., Colmegna F., Clerici M. (2022). Disentangling Binge Eating Disorder and Food Addiction: A Systematic Review and Meta-Analysis. Eat. Weight Disord. Stud. Anorex. Bulim. Obes..

[21] Imperatori C., Innamorati M., Lamis D.A., Contardi A., Continisio M., Castelnuovo G., Manzoni G.M., Fabbricatore M. (2016). Factor Structure of the Binge Eating Scale in a Large Sample of Obese and Overweight Patients Attending Low Energy Diet Therapy. Eur. Eat. Disord. Rev..

[22] Gearhardt A.N., Corbin W.R., Brownell K.D. (2009). Preliminary Validation of the Yale Food Addiction Scale. Appetite.

[23] Gearhardt A.N., Corbin W.R., Brownell K.D. (2016). Development of the Yale Food Addiction Scale Version 2.0. Psychol. Addict. Behav..

[24] Schulte E.M., Gearhardt A.N. (2017). Development of the Modified Yale Food Addiction Scale Version 2.0. Eur. Eat. Disord. Rev..

[25] Meule A., Gearhardt A.N. (2019). Ten Years of the Yale Food Addiction Scale: A Review of Version 2.0. Curr. Addict. Rep..

[26] American Psychiatric Association (2013). Diagnostic and Statistical Manual of Mental Disorders.

[27] Schroder R., Sellman J.D., Adamson S. (2017). Development and Validation of a Brief Measure of Eating Compulsivity (MEC). Subst. Use Misuse.

[28] Rossi A.A., Pietrabissa G., Gearhardt A.N., Musetti A., Castelnuovo G., Mannarini S. (2023). Eating Compulsivity in Inpatients with Severe Obesity and the General Population: The Italian Version of the Measure of Eating Compulsivity (MEC10-IT). Nutrients.

[29] Netemeyer R.G., Bearden W.O., Sharma S. (2003). Scaling Procedures: Issues and Applications.

[30] Campbell D.T., Fiske D.W. (1959). Convergent and Discriminant Validation by the Multitrait-Multimethod Matrix. Psychol. Bull..

[31] Roemer E., Schuberth F., Henseler J. (2021). HTMT2–an Improved Criterion for Assessing Discriminant Validity in Structural Equation Modeling. Ind. Manag. Data Syst..

[32] McDonald R.P. (1985). Factor Analysis and Related Methods.

[33] Rönkkö M., Cho E. (2022). An Updated Guideline for Assessing Discriminant Validity. Organ. Res. Methods.

[34] Gelman A. More on Replication Crisis. https://statmodeling.stat.columbia.edu/2016/03/03/more-on-replication-crisis/.

[35] Loken E., Gelman A. (2017). Measurement Error and the Replication Crisis. Science.

[36] Flake J.K., Fried E.I. (2020). Measurement Schmeasurement: Questionable Measurement Practices and How to Avoid Them. Adv. Methods Pract. Psychol. Sci..

[37] Gonzalez O., MacKinnon D.P., Muniz F.B. (2021). Extrinsic Convergent Validity Evidence to Prevent Jingle and Jangle Fallacies. Multivar. Behav. Res..

[38] Shaffer J.A., DeGeest D., Li A. (2016). Tackling the Problem of Construct Proliferation: A Guide to Assessing the Discriminant Validity of Conceptually Related Constructs. Organ. Res. Methods.

[39] Henseler J., Ringle C.M., Sarstedt M. (2015). A New Criterion for Assessing Discriminant Validity in Variance-Based Structural Equation Modeling. J. Acad. Mark. Sci..

[40] Fornell C., Larcker D.F. (1981). Evaluating Structural Equation Models with Unobservable Variables and Measurement Error. J. Mark. Res..

[41] Rossi A., Mannarini S., Castelnuovo G., Pietrabissa G. (2023). Disordered Eating Behaviors Related to Food Addiction/Eating Addiction in Inpatients with Obesity and the General Population: The Italian Version of the Addiction-like Eating Behaviors Scale (AEBS-IT). Nutrients.

[42] Ricca V., Mannucci E., Moretti S., Di Bernardo M., Zucchi T., Cabras P.L., Rotella C.M. (2000). Screening for Binge Eating Disorder in Obese Outpatients. Compr. Psychiatry.

[43] Marcus M.D., Wing R.R., Hopkins J. (1988). Obese Binge Eaters: Affect, Cognitions, and Response to Behavioral Weight Control. J. Consult. Clin. Psychol..

[44] Duarte C., Pinto-Gouveia J., Ferreira C. (2015). Expanding Binge Eating Assessment: Validity and Screening Value of the Binge Eating Scale in Women from the General Population. Eat. Behav..

[45] Gormally J., Black S., Daston S., Rardin D. (1982). The Assessment of Binge Eating Severity among Obese Persons. Addict. Behav..

[46] Freitas S.R., Lopes C.S., Appolinario J.C., Coutinho W. (2006). The Assessment of Binge Eating Disorder in Obese Women: A Comparison of the Binge Eating Scale with the Structured Clinical Interview for the DSM-IV. Eat. Behav..

[47] Grupski A.E., Hood M.M., Hall B.J., Azarbad L., Fitzpatrick S.L., Corsica J.A. (2013). Examining the Binge Eating Scale in Screening for Binge Eating Disorder in Bariatric Surgery Candidates. Obes. Surg..

[48] Imperatori C., Fabbricatore M., Lester D., Manzoni G.M., Castelnuovo G., Raimondi G., Innamorati M. (2019). Psychometric Properties of the Modified Yale Food Addiction Scale Version 2.0 in an Italian Non-Clinical Sample. Eat. Weight Disord. Stud. Anorex. Bulim. Obes..

[49] Pursey K.M., Gearhardt A.N., Burrows T.L. (2016). The Relationship between “Food Addiction” and Visceral Adiposity in Young Females. Physiol. Behav..

[50] R Core Team (2023). R: A Language and Environment for Statistical Computing.

[51] RStudio Team (2023). RStudio: Integrated Development for R.

[52] Revelle W. (2015). Package “psych”—Procedures for Psychological, Psychometric and Personality Research.

[53] Wickham H., Francois R., Henry L., Müller K., Vaughan D. (2023). Dplyr: A Grammar of Data Manipulation.

[54] Wickham H. (2007). Reshaping Data with the Reshape Package. J. Stat. Softw..

[55] Wickham H. (2016). Ggplot2: Elegant Graphics for Data Analysis.

[56] Heinzen E., Sinnwell J., Atkinson E., Gunderson T., Dougherty G., Votruba P., Lennon R., Hanson A., Goergen K., Lundt E. (2021). Arsenal: An Arsenal of “R” Functions for Large-Scale Statistical Summaries.

[57] Rosseel Y. (2012). Lavaan: An R Package for Structural Equation Modeling. J. Stat. Softw..

[58] Jorgensen T.D. (2018). Package “semTools”. Useful Tools for Structural Equation Modeling.

[59] Epskamp S. (2015). semPlot: Unified Visualizations of Structural Equation Models. Struct. Equ. Model. Multidiscip. J..

[60] Bryer J., Speerschneider K. (2016). Likert: Analysis and Visualization Likert Items.

[61] Flora D.B. (2020). Your Coefficient Alpha Is Probably Wrong, but Which Coefficient Omega Is Right? A Tutorial on Using R to Obtain Better Reliability Estimates. Adv. Methods Pract. Psychol. Sci..

[62] Lord F.M., Novick M.R., Birnbaum A. (1968). Statistical Theories of Mental Test Scores.

[63] Sijtsma K. (2009). On the Use, the Misuse, and the Very Limited Usefulness of Cronbach’s Alpha. Psychometrika.

[64] Woodhouse B., Jackson P.H. (1977). Lower Bounds for the Reliability of the Total Score on a Test Composed of Non-Homogeneous Items: II: A Search Procedure to Locate the Greatest Lower Bound. Psychometrika.

[65] Brown T.A. (2015). Confirmatory Factor Analysis for Applied Research.

[66] Hoyle R.H. (2012). Handbook of Structural Equation Modeling.

[67] Kline P. (2015). Introduction to Psychometric Design.

[68] Lionetti F., Keijsers L., Dellagiulia A., Pastore M. (2016). Evidence of Factorial Validity of Parental Knowledge, Control and Solicitation, and Adolescent Disclosure Scales: When the Ordered Nature of Likert Scales Matters. Front. Psychol..

[69] Muthén L.K., Muthén B.O. (1998). Mplus User’s Guide.

[70] Hu L.T., Bentler P.M. (1999). Cutoff Criteria for Fit Indexes in Covariance Structure Analysis: Conventional Criteria versus New Alternatives. Struct. Equ. Model. Multidiscip. J..

[71] Muthén B.O., Asparouhov T. (2002). Latent Variable Analysis with Categorical Outcomes: Multiple-Group And Growth Modeling In Mplus. Mplus Web Notes.

[72] Rossi A.A., Pietrabissa G., Tagliagambe A., Scuderi A., Montecchiani L., Castelnuovo G., Mannarini S., Dalla Ragione L. (2023). Many Facets of Eating Disorders: Profiling Key Psychological Features of Anorexia Nervosa and Binge Eating Disorder. Behav. Sci..

[73] Fitzpatrick R. (2000). Measurement Issues in Health-Related Quality of Life: Challenges for Health Psychology. Psychol. Health.

[74] Bottesi G., Spoto A., Freeston M.H., Sanavio E., Vidotto G. (2015). Beyond the Score: Clinical Evaluation through Formal Psychological Assessment. J. Pers. Assess..

[75] Panzeri A., Komici K., Cerutti P., Sacco D., Pistono M., Ferrario S.R. (2021). Gender Differences and Long-Term Outcome of over 75 Elderlies in Cardiac Rehabilitation: Highlighting the Role of Psychological and Physical Factors through a Secondary Analysis of a Cohort Study. Eur. J. Phys. Rehabil. Med..

[76] Bastianelli A., Spoto A., Vidotto G. (2011). Social Network Analysis and Eating Disorders: A Study Concerning Blogs. G. Ital. Med. Lav. Ergon..

[77] Panzeri A., Bettinardi O., Bottesi G., Bertolotti G., Brambatti L., Monfredo M., Mignemi G., Bruno G., Vidotto G., Spoto A. (2022). Assessment of Perceived Support in the Context of Emergency: Development and Validation of the Psycho-Social Support Scale. Curr. Psychol..

[78] Bennett K.M., Panzeri A., Derrer-Merk E., Butter S., Hartman T.K., Mason L., McBride O., Murphy J., Shevlin M., Gibson-Miller J. (2023). Predicting Resilience during the COVID-19 Pandemic in the United Kingdom: Cross-Sectional and Longitudinal Results. PLoS ONE.

[79] Flake J.K., Pek J., Hehman E. (2017). Construct Validation in Social and Personality Research: Current Practice and Recommendations. Soc. Psychol. Personal. Sci..

[80] Beleigoli A.M., Andrade A.Q., Cançado A.G., Paulo M.N., Diniz M.D.F.H., Ribeiro A.L. (2019). Web-Based Digital Health Interventions for Weight Loss and Lifestyle Habit Changes in Overweight and Obese Adults: Systematic Review and Meta-Analysis. J. Med. Internet Res..

[81] Moghimi E., Davis C., Rotondi M. (2021). The Efficacy of eHealth Interventions for the Treatment of Adults Diagnosed with Full or Subthreshold Binge Eating Disorder: Systematic Review and Meta-Analysis. J. Med. Internet Res..

[82] Moravcová K., Karbanová M., Bretschneider M.P., Sovová M., Ožana J., Sovová E. (2022). Comparing Digital Therapeutic Intervention with an Intensive Obesity Management Program: Randomized Controlled Trial. Nutrients.

[83] Panzeri A., Rossi Ferrario S. Supporting Rehabilitation Patients with COVID-19 during the Pandemic: Experiences from a Technology-Based Psychological Approach. Proceedings of the CEUR Workshop Proceedings: Second Symposium on Psychology-Based Technologies—Psychobit.

[84] Rossi Ferrario S., Panzeri A., Pistono M., Ferrario S.R., Panzeri A., Pistono M. (2021). Psychological Difficulties of LVAD Patients and Caregivers: A Follow up over 1 Year from Discharge. Artif. Organs.

[85] Heggestad E.D., Banks George C., Monroe Hausfeld M., Tonidandel S., Williams E.B. (2019). Scale Adaptation in Organizational Science Research: A Review and Best-Practice Recommendations. J. Manag..

